# Supplementary material for: Spatial patterns of species richness and nestedness in ant assemblages along an elevational gradient in a Mediterranean mountain range
Source: PLoS One. 2018 Dec 19;13(12):e0204787. doi: 10.1371/journal.pone.0204787 (PMC6300198; doi:10.1371/journal.pone.0204787)
Supplement: S2 Table — Guadarrama range (-G2014- year 2014: 18 grasslands, 35 species; -G2015- year 2015: 6 grasslands, 26 species) and Serrota range (-S2015- year 2015: 6 grasslands, 20 species). Indices are Nestedness metric based on Overlap and Decreasing Fill (NODF), NODFr for rows (i.e., localities) and NODFc for columns (i.e., species) and matrix temperature (T). Numbers under headings for indices provide index values followed by P-values estimated by equiprobable (‘r00’) binary null models and proportional resampling (‘quasiswap’). Significant P-values (P < 0.05) are in bold face. Fill: matrix fill (sum of 1s/sum of cells). (PDF) [file pone.0204787.s002.pdf]

S2 Table. Nestedness indices for dry grassland ant assemblages in surveys from all study areas in central Spain.

Grassland	NODF	NODFr	NODFc	T	fill
G2014<br><br>(18x35)	38.31:<br><br>**0.001-0.021**	40.62:<br><br>**0.001-0.001**	37.72:<br><br>**0.001**-0.143	25.25:<br><br>**0.001**-0.892	0.23
G2015<br><br>(6x26)	35.57:<br><br>**0.001**-0.445	39.7:<br><br>0.081-0.075	35.38:<br><br>**0.001**-0.383	29.16:<br><br>0.576-0.493	0.28
S2015<br><br>(6x20)	50.64:<br><br>**0.005-0.001**	55.43:<br><br>**0.021**-0.153	50.26:<br><br>**0.007-0.001**	28.66:<br><br>0.085-**0.043**	0.39

Guadarrama range (-G2014- year 2014: 18 grasslands, 35 species; -G2015- year 2015: 6 grasslands, 26 species) and Serrota range (-S2015- year 2015: 6 grasslands, 20 species). Indices are Nestedness metric based on Overlap and Decreasing Fill (NODF), NODFr for rows (i.e., localities) and NODFc for columns (i.e., species) and matrix temperature (T). Numbers under headings for indices provide index values followed by *P*-values estimated by equiprobable (‘r00’) binary null models and proportional resampling (‘quasiswap’). Significant *P*-values ($P < 0.05$) are in bold face. Fill: matrix fill (sum of 1s/sum of cells).
