# Supplementary material for: Spatial patterns of species richness and nestedness in ant assemblages along an elevational gradient in a Mediterranean mountain range
Source: PLoS One. 2018 Dec 19;13(12):e0204787. doi: 10.1371/journal.pone.0204787 (PMC6300198; doi:10.1371/journal.pone.0204787)
Supplement: S3 Table — Multiple-site dissimilarities accounting for the spatial turnover (βSIM) and the nestedness components (βNES) of beta diversity, and sum of both values (βSOR) for dry grassland ant assemblages in all study areas in central Spain. G2014: Guadarrama range 2014; G2015: Guadarrama range 2015; S2015: Serrota range 2015. P-values estimated by equiprobable binary null models (‘r00’) are given between parentheses. Significant P-values (P < 0.05) are in bold face. (PDF) [file pone.0204787.s003.pdf]

S3 Table. Multiple-site dissimilarities of beta diversity.

Grassland	β_{SOR}	β_{SIM}	β_{NES}
G2014	0.88 (**0.001**)	0.79 (**0.001**)	0.09 (**0.001**)
G2015	0.82 (0.1089)	0.69 (0.1089)	0.13 (**0.007**)
S2015	0.73 (0.6364)	0.48 (**0.001**)	0.25 (**0.001**)

Multiple-site dissimilarities accounting for the spatial turnover (β_{SIM}) and the nestedness components (β_{NES}) of beta diversity, and sum of both values (β_{SOR}) for dry grassland ant assemblages in all study areas in central Spain. G2014: Guadarrama range 2014; G2015: Guadarrama range 2015; S2015: Serrota range 2015. *P*-values estimated by equiprobable binary null models ('r00') are given between parentheses. Significant *P*-values ($P < 0.05$) are in bold face.
