# Supplementary figures and images for: Spatial patterns of species richness and nestedness in ant assemblages along an elevational gradient in a Mediterranean mountain range
Source: PLoS One. 2018 Dec 19;13(12):e0204787. doi: 10.1371/journal.pone.0204787 (PMC6300198; doi:10.1371/journal.pone.0204787)

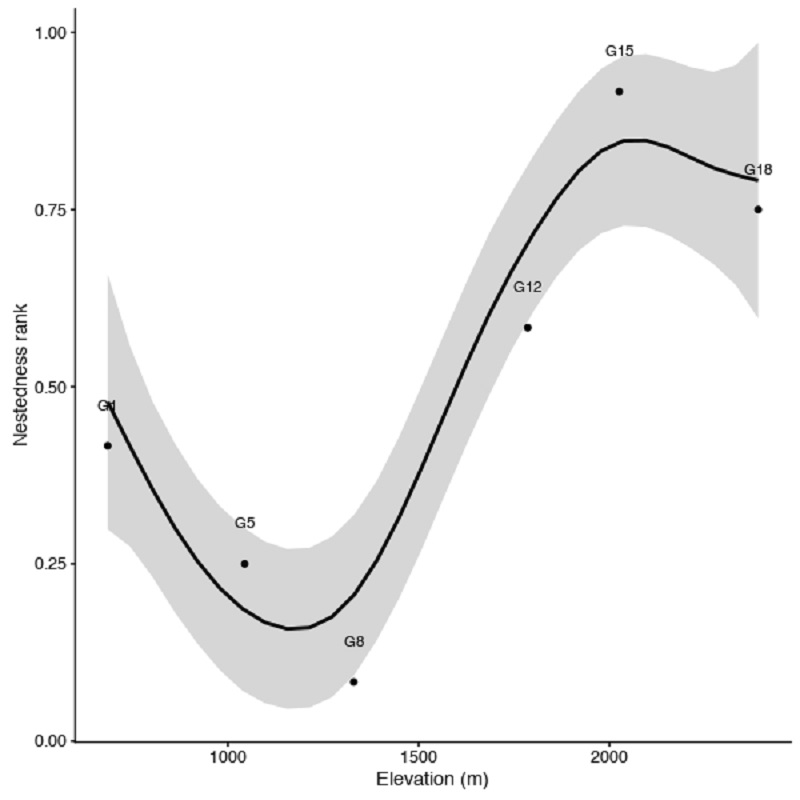

Supplement: S1 Fig — Generalized additive model (GAM) of nestedness ranks (based on nestedness temperature index) on elevation in ant assemblages from Guadarrama range (central Spain) fit to Guadarrama 2014 data and validated with Guadarrama 2015 data. (TIF) [file pone.0204787.s004.tif]

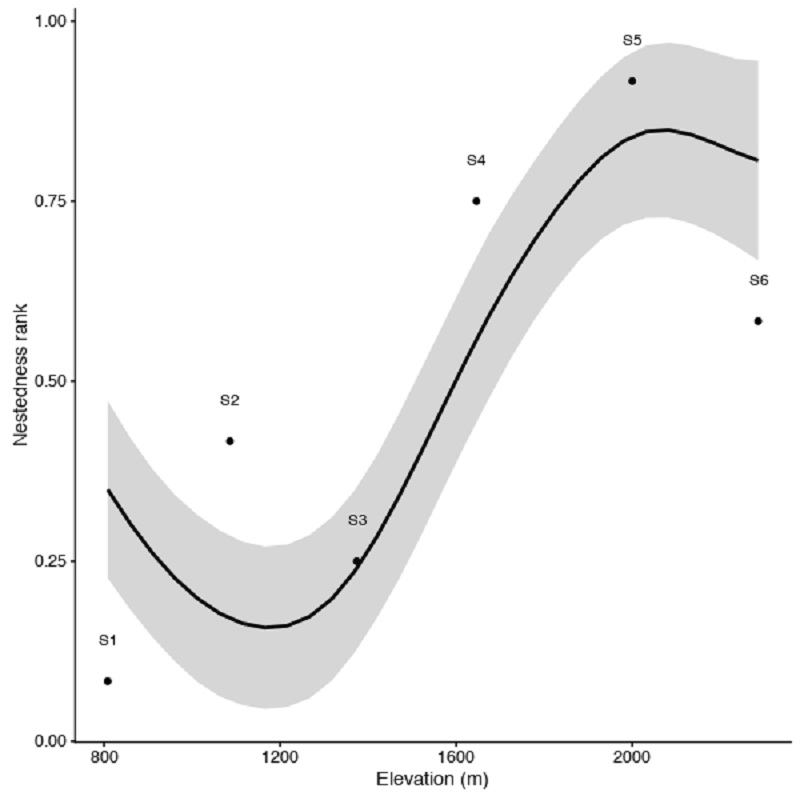

Supplement: S2 Fig — Generalized additive model (GAM) of nestedness ranks (based on nestedness temperature index) on elevation in ant assemblages from Guadarrama range (central Spain) fit to Guadarrama 2014 data and validated with Serrota 2015 data. (TIF) [file pone.0204787.s005.tif]

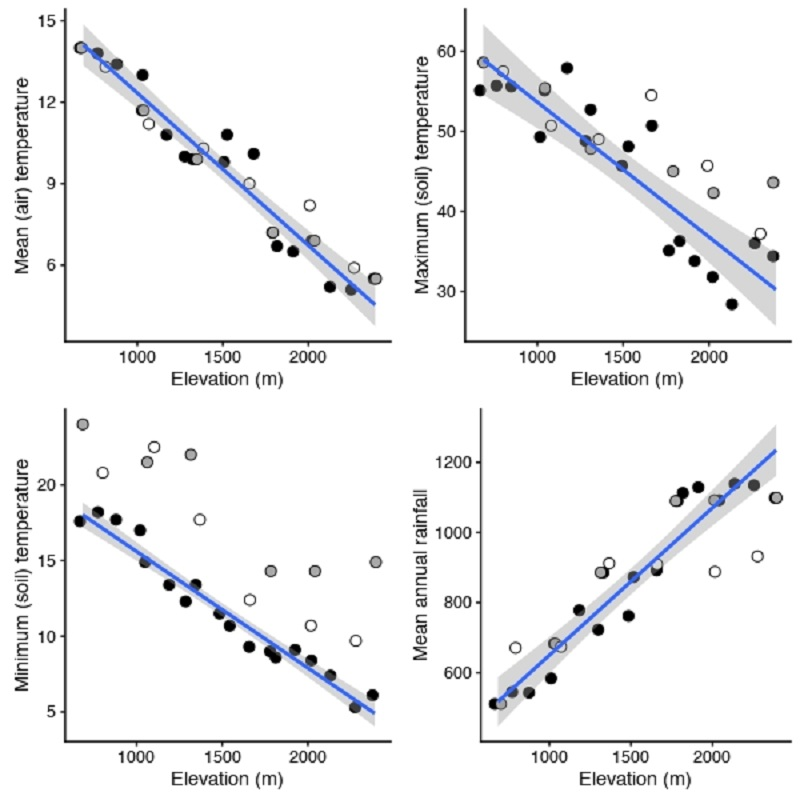

Supplement: S3 Fig — Linear regressions between elevation and mean annual (air) temperature (top left, R2 = 94%), maximum (soil) temperature (top right, R2 = 80%), minimum (soil) temperature (bottom left, R2 = 96%), and mean annual rainfall (bottom right, R2 = 91%). Regressions built for Guadarrama 2014 data (n = 18 sites, black circles). Data for Guadarrama 2015 (dark grey circles) and Serrota 2015 (white circles) are depicted as a reference. Temperatures measured in Celsius degrees and elevation in meters. Annual climatic data from the Digital Climatic Atlas of the Iberian Peninsula (http://opengis.uab.es/wms/iberia/). Soil temperatures measured for the sampling period (a week) with data loggers buried at ground level. Note that 2014 was cooler than 2015, in spite of which models for richness and nestedness built in 2014 validated well with data from 2015. (TIF) [file pone.0204787.s006.tif]
